# Leftward Lateralization of Auditory Cortex Underlies Holistic Sound Perception in Williams Syndrome

**DOI:** 10.1371/journal.pone.0012326

**Published:** 2010-08-23

**Authors:** Martina Wengenroth, Maria Blatow, Martin Bendszus, Peter Schneider

**Affiliations:** 1 Department of Neuroradiology, University of Heidelberg Medical School, Heidelberg, Germany; 2 Department of Neurology, Section of Biomagnetism, University of Heidelberg Medical School, Heidelberg, Germany; Hotchkiss Brain Institute, University of Calgary, Canada

## Abstract

**Background:**

Individuals with the rare genetic disorder Williams-Beuren syndrome (WS) are known for their characteristic auditory phenotype including strong affinity to music and sounds. In this work we attempted to pinpoint a neural substrate for the characteristic musicality in WS individuals by studying the structure-function relationship of their auditory cortex. Since WS subjects had only minor musical training due to psychomotor constraints we hypothesized that any changes compared to the control group would reflect the contribution of genetic factors to auditory processing and musicality.

**Methodology/Principal Findings:**

Using psychoacoustics, magnetoencephalography and magnetic resonance imaging, we show that WS individuals exhibit extreme and almost exclusive holistic sound perception, which stands in marked contrast to the even distribution of this trait in the general population. Functionally, this was reflected by increased amplitudes of left auditory evoked fields. On the structural level, volume of the left auditory cortex was 2.2-fold increased in WS subjects as compared to control subjects. Equivalent volumes of the auditory cortex have been previously reported for professional musicians.

**Conclusions/Significance:**

There has been an ongoing debate in the neuroscience community as to whether increased gray matter of the auditory cortex in musicians is attributable to the amount of training or innate disposition. In this study musical education of WS subjects was negligible and control subjects were carefully matched for this parameter. Therefore our results not only unravel the neural substrate for this particular auditory phenotype, but in addition propose WS as a unique genetic model for training-independent auditory system properties.

## Introduction

Musical sound perception is associated with considerable inter-individual variability. In the general population there is an even distribution between two preferential listening modes, independent of age, gender or degree of musical training: Holistic listeners perceive the sound as a whole with emphasis on the fundamental tone, whereas spectral listeners decompose the sound into its single harmonic constituents [1]. Individual sound perception also determines preference for certain musical instruments and music styles: Spectral listeners favor overtone-rich instruments (*e.g.* organ, saxophone) as well as opera and jazz music, whereas dominant holistic listeners prefer high-pitched and/or percussive instruments (*e.g.* trumpet, piano, drums) and rhythmic beats [2]. The neural substrates of individual sound perception preference were identified in the functional and morphological lateralization of the auditory cortex (AC), particularly the relative size, gyrification and shape of the Heschl's gyrus (HG) [1], [3]. However, whether such macroscopic and functional lateralization reflects genetic predisposition or training-induced neuroplasticity has been much debated in the neuroscience field and so far remained a matter of unresolved controversy.

In order to study the potential contribution of genetic factors to the auditory profile we investigated individual sound perception as well as morphology and function of the auditory cortex in subjects with Williams-Beuren syndrome (WS) and compared the findings with control subjects, who were well-matched for the amount of musical training.

WS is a rare multisystemic developmental disorder caused by a hemizygous microdeletion on chromosome 7q11.23 (OMIM #194050) [4]. Until now approximately 28 genes have been identified within this critical region and some of the WS typical features could be ascribed to the deletion of specific genes. In particular, vascular pathologies such as supravalvular aortic stenosis have been attributed to haploinsufficiency of the elastin gene (ELN). The deletion of syntaxin 1A (STX1A), LIM kinase 1 (LIMK1), CAP-GLY domain-containing linker protein 2 (CLIP2), general transcription factor II-I (GTF2I) and general transcription factor II-I repeat domain (GTF2IRD1) has been repeatedly suggested to be associated with the characteristic intellectual profile [4]–[7]. The latter includes cognitive and psychomotor deficits [8], [9], which stand in marked contrast to the unusual fascination and intense engagement with rhythm and music [10]–[12]. The vast majority of WS children participate in musical education and instrument playing; they show heightened emotional reactions to music and express high rhythmic creativity [10]–[12].

In brief, we discovered a characteristic sound perception profile in WS, associated with structural and functional augmentation of the left AC. Given that the genetic defect is well defined and musical training in affected subjects is negligible, WS offers a unique opportunity to probe training-independent auditory system properties.

## Results

### Holistic sound perception preference in WS

In order to investigate sound perception in WS we performed a standardized psychoacoustic test with 29 WS subjects and 75 healthy well-matched controls (see **Table 1** for demographic data). This test included 12 representative harmonic complex tone pairs with varying parameters of pitch and timbre (*i.e.* number, height and frequency of harmonics), which were derived from an extensive psychoacoustic test battery previously published [1] and allowed separation of dominant holistic (sound perception index δ<0) from spectral (δ≥0) listeners (see **Materials and Methods** for details). This short test version had a high correlation with the full original test (r = 0.90, *p*<0.0001), as assessed in 64 control subjects.

**Table 1:** Demographic and psychoacoustic data.

	C*_SP_*	C*_H_*	WS	*P* (WS vs. C*_SP_*)	*p* (WS vs. C*_H_*)
**Sound perception test**					
Sex (female/male)	16/11	27/21	14/15		
Age (yrs)	16.8±0.7	16.5±0.5	15.0±1.4	0.09	0.131
Musical training (hrs/d)	0.6±0.2	0.5±0.2	0.4±0.1	0.02	0.10
Sound perception index (δ)	0.61±0.05	−0.59±0.06	−0.73±0.07	<0.0001	0.03
**Neuroimaging (MEG, MRI)**					
Sex (female/male)	5/5	7/3	6/5		
Age (yrs)	18.7±1.6	18.5±1.6	17.0±2.7	0.90	0.95
Musical training (hrs/d)	0.5±0.2	0.5±0.1	0.4±0.2	0.96	0.94
Sound perception index (δ)	0.58±0.10	−0.63±0.12	−0.88±0.06	<0.0001	0.05

Sound perception: Index δ =  (*SP* - *H*)/(*SP*+*H*) according to the number of perceived holistic (*H*) and spectral (*SP*) items of the sound perception test [1]. Age (years), musical expertise (hours of training per day) and sound perception index (δ) are presented as mean ± standard error (s.e.m.). ANOVA: *p-*value of WS vs. control group of spectral listeners (C*_SP_*) and WS vs. control group of holistic listeners (C*_H_*).

**Figure 1:**
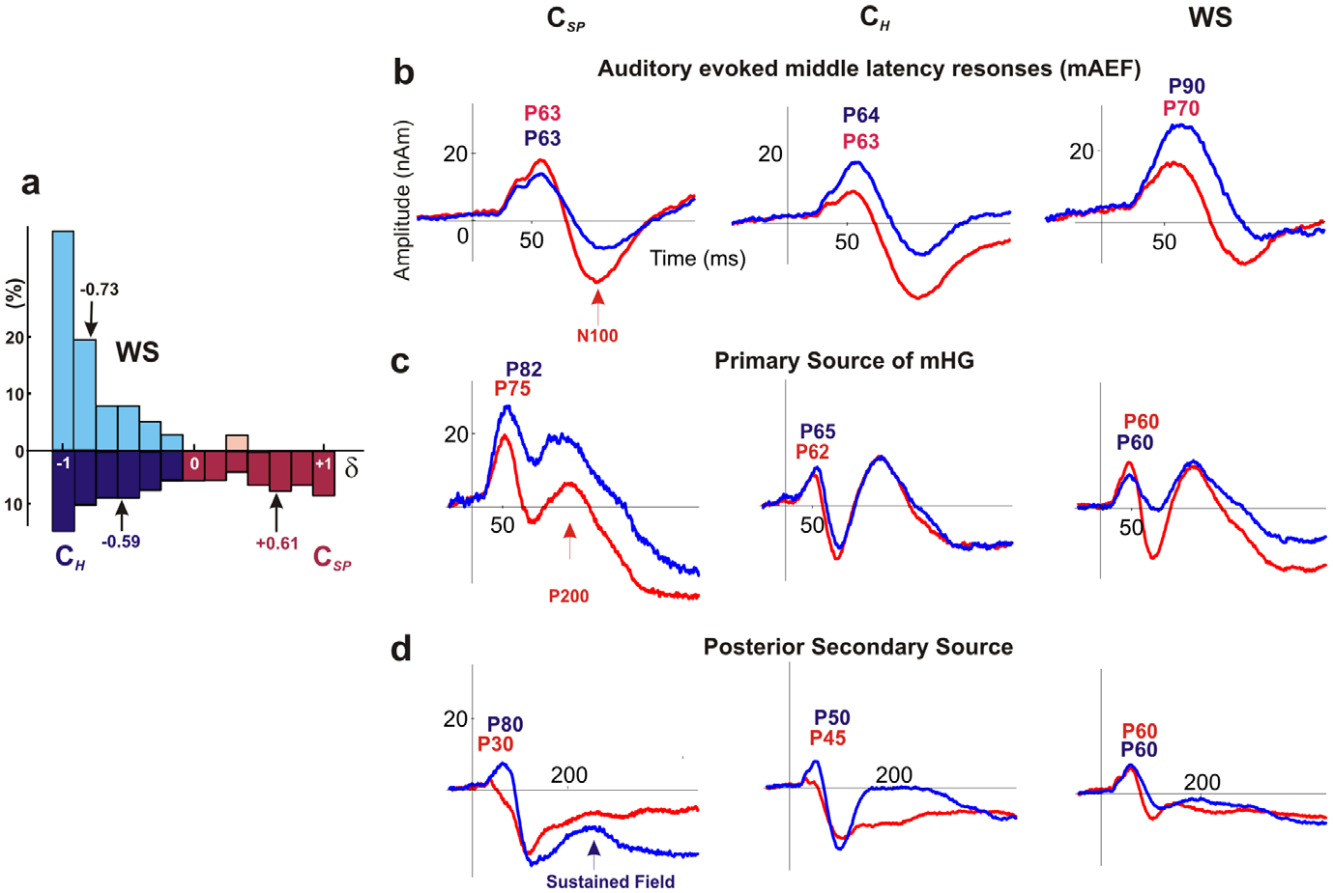
Holistic sound perception and functional leftward lateralization in WS. (**a**) Sound perception index (δ) of spectral (C*_SP_*: red) and holistic listeners (C*_H_*: blue) of the control group (dark colors) and the WS group (light colors). Mean δ of groups are indicated (arrows). (**b–d**) Averaged auditory evoked fields (AEFs) of three experimental groups: Left-hemispheric: blue, right-hemispheric: red traces. Peak latency in ms relative to tone onset. (**b**) Middle latency components of auditory evoked field (mAEF) modeled by one fixed dipole in each hemisphere at |x| = 45, time range 0–200 ms (**c–d**). Late auditory evoked field modeled by two fixed dipoles in each hemisphere, time range 0 – 500 ms (**c**) a primary source seeded in the first transverse Heschl's gyrus, and (**d**) a second source seeded in the posterior part of HG duplications.

In keeping with results of previous studies, the control group of this experiment showed an even distribution of holistic (C*_H_*) and spectral (C*_SP_*) listeners (C*_H_*: mean δ = −0.59±0.06; C*_SP_*: mean δ = 0.61±0.05; **Fig. 1a** **,** **Table 1**). In striking contrast to the even distribution of C*_H_* and C*_SP_* listeners within the control group, WS subjects showed extreme and almost exclusive holistic sound perception (all controls: mean δ = −0.11 vs. WS: mean δ = −0.73±0.07; *p*<0.0001; **Fig. 1a** **,** **Table 1**). The sound perception index was distinctly shifted towards the left end in favor of extreme holistic sound perception in WS subjects, even if compared only to the holistic listeners of the control group. Only two out of 29 WS individuals scored within the lower spectral range. The sound perception test was repeated at a later time point in 7 WS individuals; the test-retest reliability was very high (r = 0.95; *p*<0.0001). Thus, at the behavioral level WS individuals exhibited extreme holistic sound perception, representing a strong deviation from the general population.

### Left-dominant asymmetry of auditory evoked fields (AEF) in WS

Magnetoencephalography (MEG) was performed to measure immediate auditory evoked fields (AEF) during passive listening to instrumental tones. Spatiotemporal source modeling with one dipole in each hemisphere was used to calculate the auditory evoked middle latency responses (mAEF) of AC. In line with previous investigations [1], [3] a characteristic asymmetry of the mAEF peaking 50 ms after tone onset (P50) was observed in the control groups. Namely, the P50 dipole amplitudes were increased in the left AC in C*_H_* listeners, whereas C*_SP_* listeners exhibited larger right AEF, typically with multiple peaks (**Table 2**, **Fig. 1b**).

**Table 2:** MEG source activity.

	C*_SP_*	C*_H_*	WS	*p* (WS vs. C*_SP_*)	*p* (WS vs. C*_H_*)
N	10	10	9		
P50 Amplitudes (nAm)					
Right AEF	23.1±3.3	15.2±1.9	20.7±2.8	0.45	0.09
Left AEF	17.8±2.1	24.5±4.7	36.1±3.2	<0.0001	0.02
Dipole Localization (mm)					
Right hemisphere	13.2±1.7	12.3±1.8	25.5±1.7	0.0086	0.0077
Left hemisphere	19.6±1.6	22.8±1.7	29.5±1.9	0.003	0.018

Source activity: Dipole amplitudes of auditory evoked P50responses (nAm). Dipole localization: normalized y-coordinate in anterior-posterior direction (mm). ANOVA: *p-*value of WS vs. spectral listeners of control group (C*_SP_*) and WS vs. holistic listeners of control group (C*_H_*).

In a second step, source modeling with two dipoles in each hemisphere was used to separate the characteristic responses from the primary core areas of anterior Heschl's gyrus from the secondary responses of the surrounding belt areas. Characteristic differences of the primary and secondary responses were detected with respect of curve progression, amplitude, latency and width of the middle and late auditory evoked field and the superimposed sustained field components (**Fig. 1c,d**).

In the WS group, both early and late source activities were markedly lateralized to the left hemisphere, reflecting their strong holistic sound perception. Furthermore in WS subjects the response of secondary auditory cortex of the left hemisphere showed a strong sustained field component following the onset response, which was 3.1-fold larger as compared to controls (average amplitude in time range 100–500 ms: 15.4 nAm (WS) vs. 4.9 nAm (controls); **Fig. 1c,d**). This effect was not observed in the right hemisphere. Additionally, in comparison to the control group left-hemispheric dipole amplitudes were found to be increased by 103% as compared to C*_SP_* and 47% as compared to C*_H_* listeners (**Fig. 1b**, **Table 2**). It is of further note that P50 and N100 latencies were bilaterally decelerated in the WS population and not in the control group (right: P77, N135, left: P90, N175).

### Increased volume of auditory cortex and structural leftward asymmetry in WS

**Figure 2:**
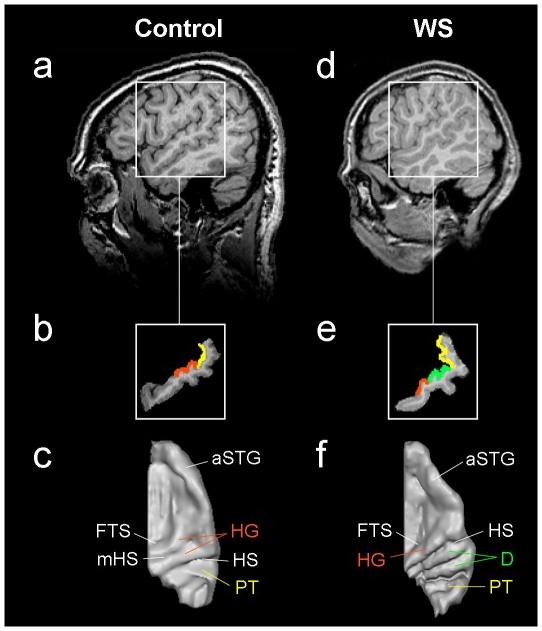
Anatomical landmarks of the auditory cortex. Auditory cortex (AC) of one control person (**a–c**) and one WS subject (**d–e**). Sagittal MR image at TAL x = 50 (**a,d**: left side of the image is the anterior part of the brain). Segmented STG (**b,e**) including Heschl's gyrus (HG; marked orange), planum temporale (PT; marked yellow) and two posterior duplications of HG in the WS subject (D; marked green). Three-dimensional surface reconstruction of right AC (**c,f**) reveals anatomical features and individual peculiarities such as D (**f**) or medial Heschl's sulcus (mHS; **c**). FTS =  first transverse sulcus; HS =  Heschl's sulcus; aSTG =  anterior superior temporal gyrus.

High-resolution magnetic resonance imaging (MRI) was performed at 3 Tesla. T1-weighted three-dimensional MR images of the brain were individually analyzed for gray matter volumes of the whole brain and the AC before and after normalization into Talairach (TAL) space (see **Materials and Methods** for details; **Fig. 2** **and** **3**).

**Figure 3:**
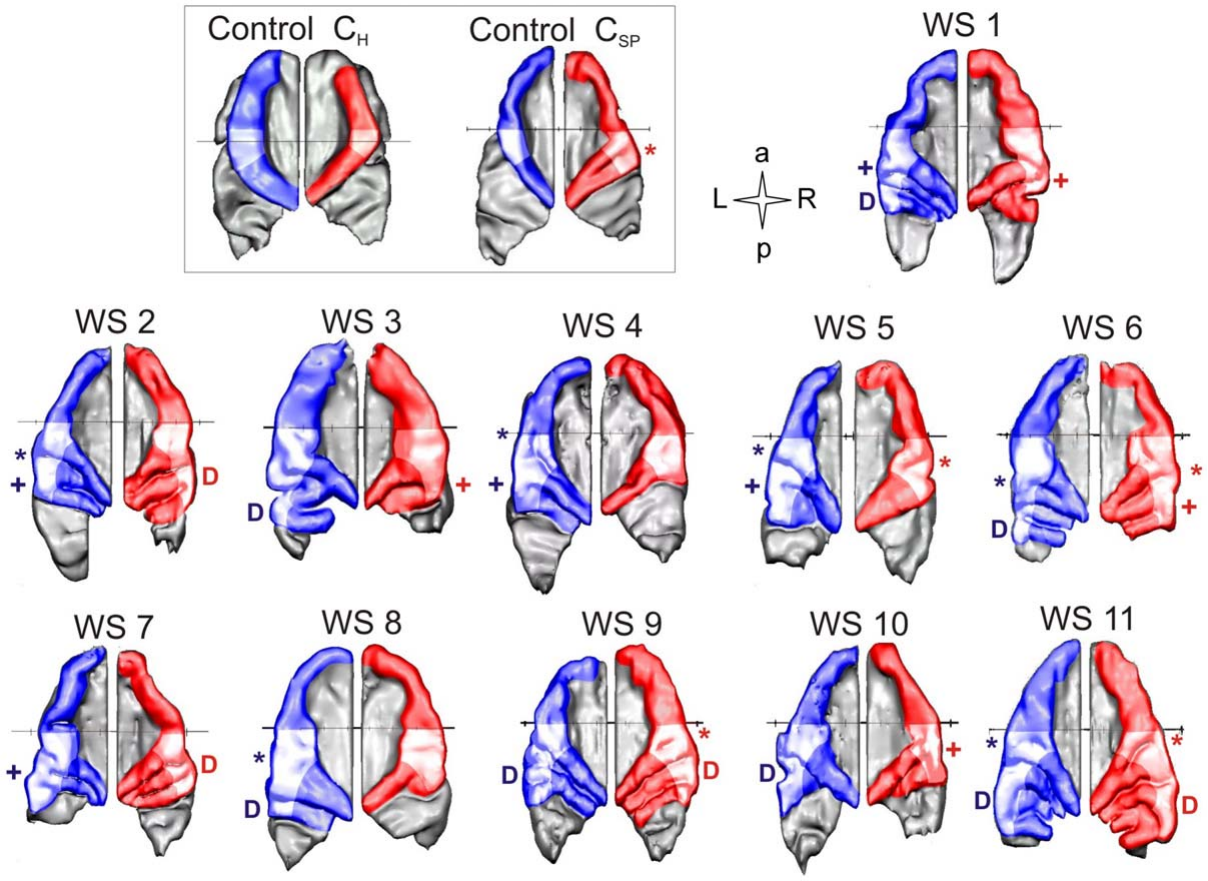
Increased gyrification of the auditory cortex in WS. Individual segmentation reveals distinct morphology of right (red) and left (blue) auditory cortex (AC) of WS subjects. In comparison, representative AC examples of one holistic listener (C_H_) and one spectral listener (C_SP_) of the control group are depicted. Lateral pitch sensitive regions of the HG are highlighted and complete posterior duplications are marked (D), if present. The position of the anterior commissure is indicated as a black line. Sulcus intermedius (*); medial duplication (+).

**Figure 4:**
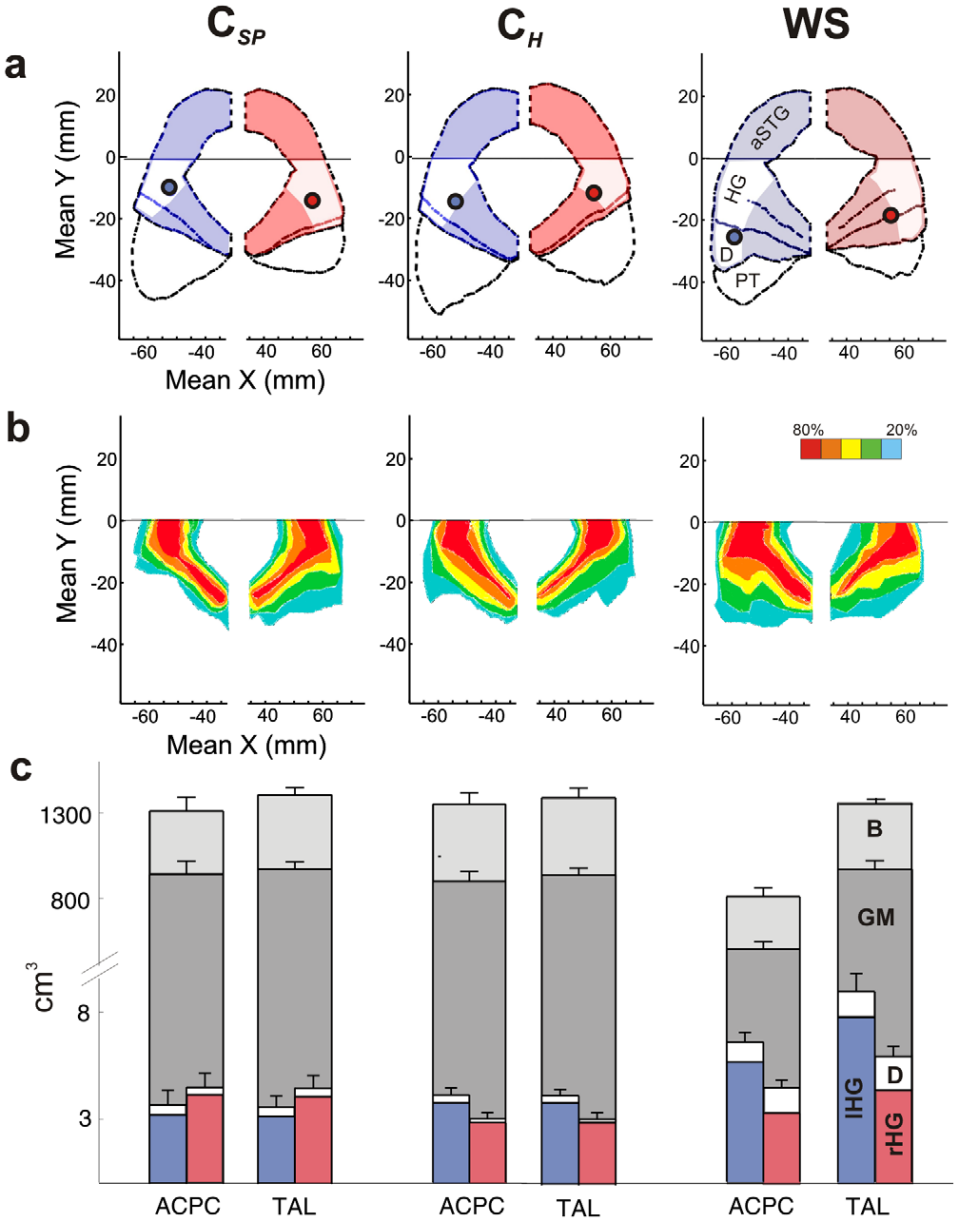
Increased gray matter volume of the auditory cortex and higher incidence of posterior duplications in WS subjects. (**a**) Averaged individual AC landmarks and MEG dipoles (filled circles) (**b**) Probability maps of HG including local duplications anterior to the first complete Heschl's sulcus. The number of overlapping voxels is color coded, *i.e.* red means that >80% of the brains overlapped in this voxel. (**a,b**) Plots in xy-Talairach (TAL) stereotaxic coordinates. (**c**) Morphometry of whole brain (B, light grey), grey matter (GM, medium grey), left HG (blue) and right HG (red) before and after normalization. ACPC =  plane of anterior and posterior commissure; a =  anterior; p =  posterior; r =  right; l =  left; aSTG =  anterior supratemporal gyrus; HG =  Heschl's Gyrus; D =  complete posterior HG duplication; PT =  planum temporale; B =  total brain volume; GM =  gray matter.

In keeping with previous volumetric studies by numerous authors [9],[13],[14],[15], the WS group in our experiment exhibited reduced total brain volume by 24%, which was primarily attributable to a disproportionate white matter reduction (**Fig. 4c** **,** **Table 3**). In contrast, the AC of WS subjects showed markedly enhanced gyrification and increased gray matter volume, regarding averaged landmarks (**Fig. 4a**), probabilistic maps (**Fig. 4b**) and individual morphology of HG (**Fig. 3** **,** **4c**).

**Table 3:** MRI morphometry.

	C*_SP_*	C*_H_*	WS	*p* (WS vs. C*_SP_*)	*p* (WS vs. C*_H_*)
N	10	10	11		
Volumes (cm^3^)					
**TAL**					
B	1358±25	1349±33	1319±21	0.25	0.45
GM	880±20	863±20	869±25	0.73	0.85
rHG	4.1±0.6	2.9±0.3	4.2±0.5	0.86	0.044
rHG incl. D	4.5±0.5	3.1±0.3	5.8±0.4	0.054	<0.0001
lHG	3.2±0.3	3.8±0.2	7.6±0.9	0.0001	0.0004
lHG incl. D	3.6±0.5	4.2±0.3	8.8±0.8	<0.0001	<0.0001
**ACPC**					
B	1298±47	1338±41	1000±32	<0.0001	<0.00001
GM	863±35	852±27	653±21	<0.0001	<0.0001
rHG	4.1±0.7	2.8±0.3	3.2±0.4	0.26	0.47
rHG incl. D	4.5±0.6	3.0±0.3	4.4±0.4	0.9	0.0068
lHG	3.2±0.40	3.8±0.21	5.6±0.4	0.0003	0.0005
lHG incl. D	3.7±0.7	4.1±0.4	6.5±0.5	0.0023	0.0006
**Frequency of D (R/L)**	3/2	1/1	5/5		

Mean volumes (cm^3^) ± s.e.m. before (in plane of anterior and posterior commissure, ACPC) and after Talairach normalization (TAL). B =  total brain volume; GM =  gray matter; rHG/lHG =  right and left Heschl's Gyrus; D =  posterior HG duplications. ANOVA: *p-*value of WS vs. C_SP_ and WS vs. C_H_.

After adjusting for total brain volume differences, HG volume was 2.2-fold increased in the left and 1.2-fold in the right hemisphere in WS as compared to control subjects. Remarkably, left HG volumes were still 1.6-fold increased in WS subjects even before taking brain volume differences into account (F_(1,19)_ = 19.59, *p*<0.0005) and the volume of the right HG matched that of the controls (**Fig. 4c** **,** **Table 3**).

Interestingly, we very often observed multiple duplications with three or four transverse HG in WS subjects, which occurs only rarely in the general population. The incidence of complete posterior HG duplications was 45% in WS compared to 18% in controls. If duplications were included in the morphometric analysis, volume changes of HG were even more pronounced (**Fig. 4c** **,** **Table 3**).

**Figure 5:**
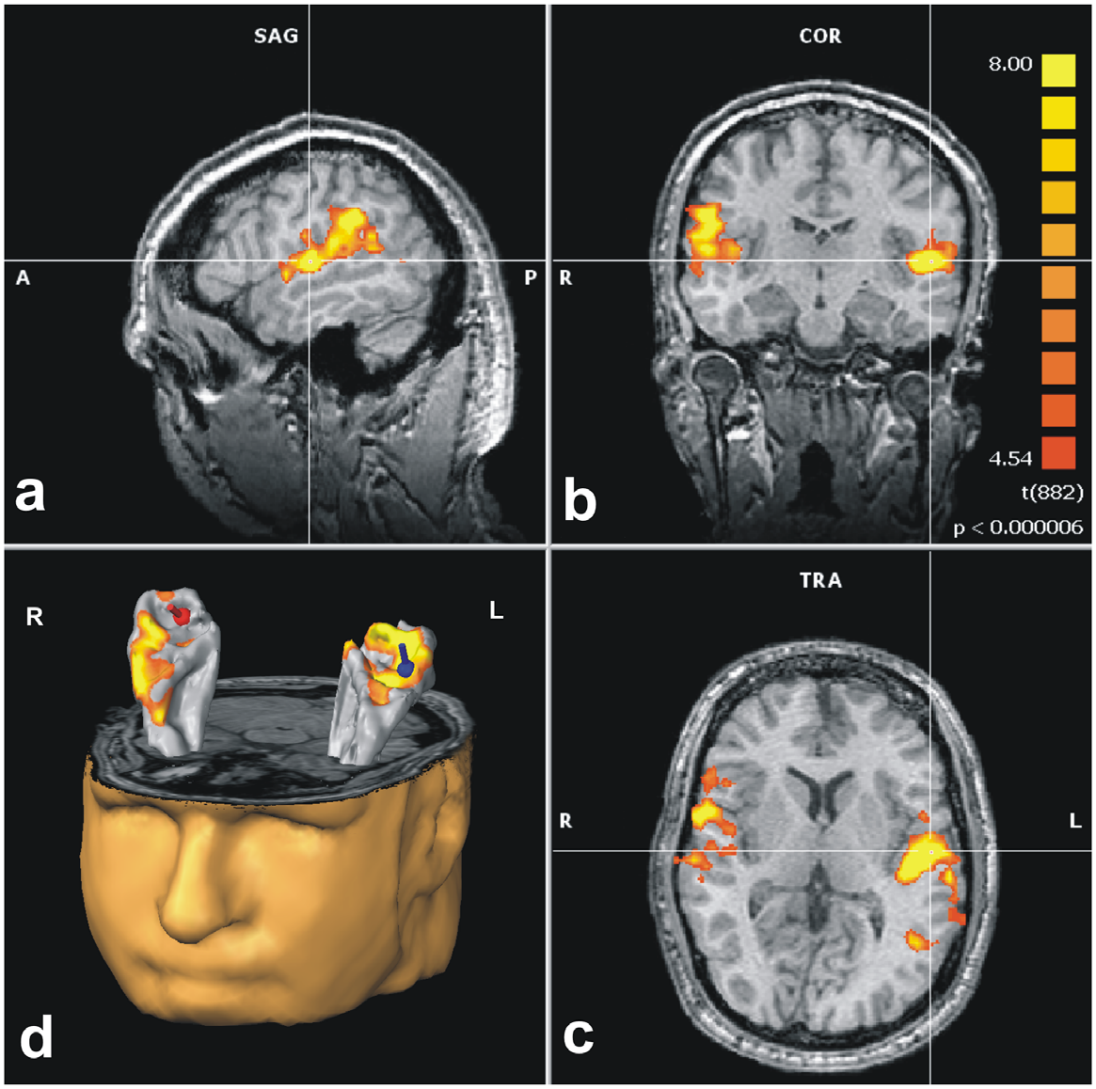
Bilateral fMRI activation of the auditory cortex including posterior duplications. Group BOLD activations of WS subjects in response to auditory stimulation are projected onto one individual WS brain in (**a**) sagittal, (**b**) coronal and (**c**) transverse planes. The cross line depicts the position of left HG. (**d**) Group BOLD-activations and MEG dipoles are projected onto an individual AC surface mesh. (A =  anterior, P =  posterior, R =  right, L =  left).

In order to probe functional significance of HG duplications, functional MRI (fMRI) with auditory stimulation using instrumental and complex tones (in analogy to the MEG stimuli employed in the study) was performed on a proof of principle basis in three WS individuals with pronounced posterior duplications. In all cases blood oxygen level dependent (BOLD) activations extended to posterior duplications, suggesting their implication in auditory processing (**Fig. 5**). In this context it is of note that the averaged MEG dipoles, which localize the center of primary activation during auditory processing, were shifted towards posterior parts of the AC by 9 mm in the left and 13 mm in the right hemisphere of WS subjects.

## Discussion

Individuals with WS are known to be mesmerized by music and to show heightened emotional reactions to sounds of all kinds [10], [11]. Their musical engagement and receptivity almost uniformly surpasses that of typically developing age peers. WS subjects also possess a remarkable ability to recognize and imitate environmental sounds, such as cars, vacuum cleaners or lawnmowers [10]. Given that the auditory phenotype is strikingly homogenous across the group and the genetic defect is well characterized, WS offers a rare opportunity to study the relationship between genes, behavior, brain morphology and function. In the present study, we employed a multimodal psychoacoustic and neuroimaging approach and found extreme holistic sound perception in WS, coupled with functional and structural leftward asymmetry of AC.

Extreme and almost uniform holistic sound perception in WS represents a significant deviation from the distribution of sound perception in the general population. Strong holistic sound perception is in agreement with the fact that the WS individuals express high rhythmic creativity [12] and are particularly fond of rhythmic and percussive instruments such as drums, keyboard and piano [16].

A number of groups have previously analyzed brain morphology in WS and consistently found total brain volume reduction, mainly attributable to reduced parietal and occipital lobe volumes [8], [15]–[19]. On the other hand increased gyrification and cortical complexity were repeatedly observed [14], [17]–[24]. Additionally, several brain areas have been reported as relatively preserved, such as superior temporal gyrus (STG), amygdala, orbitofrontal cortex and posterior vermis of the cerebellum; often accompanied by a left-hemispheric dominance [13], [15], [17]–[21], [24], [25]. The relatively preserved volumes and greater cortical complexity of structures along the Sylvian Fissure were proposed to underlie the distinctive linguistic and auditory strengths in WS [14], [18], [21]–[23]; however, this hypothesis has not been sufficiently corroborated so far.

The auditory phenotype and musical abilities in WS have been characterized by a number of investigators [9], [26], [27] and some groups have attempted to correlate the unusual auditory phenotype with a neural substrate [11], [15], [28], [29]. *Post mortem* studies on a small collection of WS brains have found preserved size of auditory cortex [30]. But MRI-based neuroimaging studies that employed group analysis methods such as voxel based morphometry (VBM) could not corroborate such findings. It is a known caveat of the VBM technique that the considerable inter-individual variability of cortical and sulcal structures might be obscured [24], [31]. Very recently Martens et al. employed an individual analysis method and found significant volume increase of the left PT in a subgroup of WS subjects who demonstrated specific musical strengths [15]; but this finding remained unexplained so far.

In the present study we applied an individual analysis method of high resolution MRI images to account for the differences of peripheral cortical structures such as the HG. In agreement with previous individual HG morphometry in musicians, HG was larger in the right hemisphere in C*_SP_* listeners of the control group and more pronounced in the left hemisphere in C*_H_* listeners. In WS subjects we found strong leftward HG asymmetry correlating with their extreme holistic sound perception. Furthermore, HG volumes exceeded by far those of normal controls, identifying a very probable neural basis of the distinctive auditory skills of WS individuals. This finding bore up against brain normalization, that is left HG volumes in WS subjects were also increased before taking total brain volume reduction into account. Such over-proportional HG volumes can be typically encountered in professional musicians and talented music students [3].

So far it remained a matter of unresolved controversy in the neuroscience community, whether such cortical volume differences in the AC reflect neuroplastic effects due to intense training [32], [33] or represent an innate predisposition to a particular talent, *i.e.* musicality. Schneider et al.’s previous work suggests that AC volume correlates with musical aptitude independent of the degree of musical training [3]. The present results corroborate this idea, since musical training was negligible in WS participants and the control subjects have been specifically matched for this parameter.

At the functional level auditory evoked fields measured by MEG showed the expected rightward asymmetry in C*_SP_* listeners and leftward asymmetry in C*_H_* listeners of the control groups. Such relative auditory lateralization based on individual sound perception has been previously reported [1] and originates from dominant processing of temporal resolution and holistic sound perception in left AC [1], [34] and spectral as well as fine pitch resolution in right AC, respectively [35]. In agreement with the psychoacoustic test results, we found a strong functional leftward asymmetry in the WS group as an electrophysiological correlate of their extreme holistic sound perception. In addition to this *relative* asymmetry, amplitudes of left AEF in WS subjects were increased in *absolute* terms to almost twice the size as compared to normal controls. Since equivalent P50 amplitudes have been reported for professional musicians [3], increased left auditory responses in WS individuals might be pointing to a putative electrophysiological substrate of their particular musicality.

In the general population the PT [36], which is the plane cortical structure posterior to the HG, is typically more extended in the left hemisphere compared to the right due to a generally smaller left HG [37], [38]. It has been independently reported by several authors that this PT asymmetry is often reduced in WS [17], [18], [22], [24]. But so far, there was no satisfactory explanation for this phenomenon. The present study is the first to identify that the reduced PT asymmetry is in fact consequential to increased left HG volume. Unfortunately, calculated HG volumes cannot necessarily be compared to all previous studies on HG volumes due to the inconsistent definition of anatomical AC landmarks. Martens et al. did not find any differences of the “primary AC” volumes but revealed increased volumes of the PT in WS in comparison to controls (as opposed to increased HG in our study). However, the authors employed different definitions of AC structures and discuss restricted comparability as a critical point. We believe that their data are well in line with ours since structures they categorized as PT (*i.e.* partial HG duplications) would have been ascribed to HG or HG duplications according to our definition. Interestingly in this respect is that Martens et al. also found leftward asymmetry (of the planum temporale) in a subgroup of particularly musical WS subjects, which is in accordance with our results [15].

We based our definitions of AC landmarks on results of cytoarchitectonic studies that demonstrated high inter-individual variability of the anatomical borders between the primary and the secondary auditory cortex [39], [40], which cannot be distinguished by morphological criteria alone. In order to estimate the borders of the primary and secondary AC we employed functional localizers and probability maps according to well-established landmarks [41]–[43]. Roughly, the primary AC is located within the medial two thirds of HG [44] and the secondary AC includes surrounding belt areas, particularly lateral areas of HG and posterior HG duplications. Evidently, consistent application of AC structures would facilitate the comparability of data across studies and would be desirable in the future.

A further remarkable finding in the present study was increased gyrification of the HG, *i.e.* higher occurrence of complete posterior HG duplications. If these were included into morphometric analysis, volume changes of HG were even more pronounced. The role of posterior HG duplications has not been sufficiently addressed in the literature as yet. An increased incidence of HG duplications has been reported in subjects with dyslexia [45] but remained unexplained so far. Whether to attribute such duplications to the HG or to be part of the planum temporale is discussed controversially in the field [15], [46]. In our study, the dorsal shift of averaged MEG dipoles and the localization of BOLD-activations indicated that HG duplications were implicated in early auditory processing. Further investigations on a larger sample set are certainly warranted in order to fully understand the structure-function relationship of HG and its duplications and are currently underway in our laboratory.

A limitation of our study is the relatively small sample size for neuroimaging tests, which is mainly attributable to low incidence of WS, reduced attention span of subjects and frequent MRI contraindication (because of *e.g.* aortic valve prostheses or pacemakers). In addition we applied an early age cut-off in order not to interfere with age related brain volume reduction. WS subjects were recruited over a period of more than two years. However, our data showed very low variance and the group was extremely homogenous. Due to the hypothesis-driven approach, individual analysis method (not group averages) and homogenous data we are confident that the number is sufficient to validate our results.

In brief, we propose WS as a unique genetic model to investigate training-independent auditory properties. Additional studies which take candidate genes from the WS critical region into consideration will lead the way to extend our understanding of the genetic influence on musicality.

## Materials and Methods

### Participants

Children and adolescents with a clinical diagnosis of WS were recruited on seminars for affected families and via an article on the German Williams syndrome website [47] over a period of more than two years. In order to avoid potential confounds with age related brain volume reduction only subjects under the age of 39 years were included.

A group of 36 WS subjects participated in a psychoacoustic sound perception test. In 7 WS subjects who were not able to fully comprehend the psychoacoustic test, the sound perception data could not be analyzed (not included in **Table 1**). A subgroup of 12 WS individuals participated in further neuroimaging studies including magnetic resonance imaging (MRI) and magnetoencephalography (MEG). Two participants had to be excluded from MEG analysis due to severe metal artifacts caused by dental braces. The MR image quality of one WS child (who was able to perform MEG) was too poor for further analysis due to movement artifacts. In 3 WS individuals it was possible to obtain functional MRI (fMRI) with auditory stimulation.

For psychoacoustic testing and neuroimaging, 20 control subjects were matched group-wise for sex, chronological age and daily hours of musical training. The control group contained 6 unaffected brothers and sisters of WS subjects. To increase statistical power of the differences found in the sound perception test, we included psychoacoustic data of 64 participants, which we selected from a previous study according to comparable demographic characteristics [1]. Control subjects were separated by their sound perception index (δ) into dominant spectral (C*_SP_*) or holistic listeners (C*_H_*). Demographic data are summarized in **Table 1**. Experimental procedures were approved by the Ethics committee of the University of Heidelberg and all participants (or parents, respectively) provided written informed consent.

### Sound perception test

We tested dominant sound perception with a representative subset of 12 tone pairs selected from an extensive psychoacoustic test previously published [1]. This short test version of three minutes duration required only a short attention span and was therefore well applicable for WS subjects and children. Each tone pair consisted of two consecutive harmonic complex tones (duration 500 ms, 10 ms rise/fall time, inter-stimulus interval 250 ms). The test tones varied in number (2, 3, 4), height (low or high partials in relation to the fundamental) and averaged frequency of harmonics (low = 0.8, high = 1.5 kHz). Parameters of harmonics which characterize timbre (*e.g.* upper component frequency) were deliberately kept constant within a tone pair to minimize timbre changes. Subjects had to decide in a two-way forced choice task whether they perceived the second tone of a tone pair as higher or lower compared to the first. Alternatively, children could sing or hum the perceived sounds. The perceived direction of the tone shift was upward or downward, depending on the subject's dominant spectral (*SP*) or holistic (*H*) sound perception. An index of sound perception preference was then computed according to the number of *SP* versus *H* classifications, using the formula δ = (*SP* - *H*)/(*SP*+*H*). Accordingly, subjects were grouped into dominant spectral listeners (δ≥0) or holistic listeners (δ<0) [1], [2]. It is of note that the “holistic” listening mode was previously referred to as “fundamental” listening mode for reason of precise terminology at the time, since only the pitch component of sound perception was studied (independent of timbre differences) [1], [2]. Statistical significance between groups was assessed using analysis of variance (ANOVA, significance level *p*<0.05).

### Magnetoencephalography (MEG)

Auditory evoked fields (AEFs) were recorded in response to characteristic instrumental tones (*e.g.* piano, organ, guitar, percussion, voice) and complex tones employing a Neuromag-122 whole-head MEG system. Subjects were instructed to passively listen to the sounds (total average of 900 instrumental and complex tones in pseudo-randomized order, tone length 500 ms, inter-stimulus interval range 400–600 ms). Cortical responses were individually analyzed with BESA program (MEGIS Software GmbH, Graefelfing) employing different models. In a first step the source activity of the primary and secondary auditory cortex was modeled with one equivalent dipole in each hemisphere to separate the early P30–P50 response complex peaking 30–50 ms after tone onset from the later N100 response (**Fig. 1b**). A combined fit-seeding technique was employed with a fixed depth value of |x| = 45 mm, because the dipole depth is the weakest parameter in MEG dipole fitting and has a strong inverse correlation with dipole amplitude [1], [3], [48]. Signal strength was calculated for each peak relative to a 100 ms baseline. Latencies and amplitudes of the P50 (**Table 2**) were analyzed on an individual level and then averaged across groups. Dipole localization was determined averaging P50 responses to all auditory stimuli (**Table 2** **,** **Fig. 4a**). Statistical significance between groups was assessed using analysis of variance and multiple analyses of variance (ANOVA and MANOVA, significance level *p*<0.05). In a second step we used spatiotemporal source modeling with two dipoles in each hemisphere to separate the characteristic responses from the primary core areas of anterior Heschl's gyrus from the secondary responses of the surrounding belt areas [3]: the first dipole within the center of the first HG (≈ primary AC, stereotactic Talairach coordinates x = +/−45, y = −5, z = 10) and the second dipole in the PT or the postero-lateral part of HG duplications, respectively (if present) (≈ secondary AC, coordinates x = +/−55, y = −30, z = 10). and fitted the orientation of the dipoles in all subjects. The resulting primary and secondary source waveforms are shown in **Fig. 1c,d**.

### Magnetic resonance imaging (MRI) and morphometry

High-resolution T1-weighted three-dimensional MR images of the brain (magnetization-prepared rapid acquisition of gradient echo (MPRAGE) sequence: echo time  = 3.47 ms, repetition time  = 1930 ms, 1 mm^3^ isotropic resolution, flip angle 15°, 176 contiguous sagittal slices, matrix size 256 mm) were acquired at 3 Tesla (Magnetom, Siemens, Erlangen, Germany) with an 8-channel head coil. Additional T2-weighted sequences were obtained and assessed by a neuroradiologist (blind to diagnosis) for potential non-WS-related pathologies. MR morphometry was performed using semi-automated BrainVoyager segmentation software (Brain Innovation QX version 1.8). Images were corrected for inhomogeneity, transformed into anterior commissure-posterior commissure plane (ACPC) and subsequently normalized in Talairach space (TAL). Subsequent to removal of non-brain tissue from the images (*i.e.*, meninges, orbits), segmentation of the whole brain and three-dimensional surface reconstruction of auditory cortices was performed in standard ACPC space and after TAL normalization. To compare anatomical landmarks between groups, stereotaxic Talairach (TAL) coordinates of individual AC were mapped and then plotted group-wise for comparison using Matlab (Mathworks, Natick, MA) (**Fig. 4a**). Probability maps of HG including local duplications anterior to the first complete Heschl's sulcus have been calculated. The number of overlapping voxels was color coded, *i.e.* red voxel represent >80% overlap of all brains under investigation (**Fig. 4b**).

Volumes of whole brain as well as left and right HG (with and without complete posterior duplications, if present) were determined according to individual intensity histograms (**Fig. 2** **,** **3** **,** **4c**) with a voxel-counting algorithm. The whole brain was defined as gray and white matter of the cerebrum, brain stem and cerebellum with the inferior boundary being the caudal end of cerebellar tonsils. Cerebrospinal fluid was not included into the calculation.

The supratemporal cortex (STG) was segmented on sagittal images in a semi-automated slice-by-slice approach according to established criteria [1], [3] including Heschl's gyrus (HG), anterior supratemporal cortex (aSTG) and planum temporale (see **Fig. 2**). The HG is the most anterior transverse gyrus of STG located between the first transverse sulcus (FTS) and Heschl's sulcus (HS). In case of multiple gyration, transverse gyri posterior to the first HG were considered posterior duplications (D, **Fig. 2e,f**) if they were separated from HG by a complete HS. Often (but inconsistently) HG was indented by a local sulcus in its central, lateral or medial part (*i.e.* medial Heschl's sulcus, mHS as in **Fig. 2c**). For morphometric analysis, the subdivided HG was calculated including its various medial or lateral duplications anterior to the first complete HS. Occurring complete posterior duplications of HG were evaluated separately in morphometric and functional analysis in order to address the current controversy on their functional significance.

The PT is the plane cortical structure posterior to the HG. Its anterior border is the complete HS posterior to HG (**Fig. 2b,c**). In case of multiple complete posterior duplications the anterior border of PT was defined as the last complete transverse sulcus posterior to the duplications (**Fig. 2e,f**). The posterior border of PT was defined as the origin of the ascending ramus (if present), the medial border was the insular cortex and the inferior border was the supratemporal sulcus [49].

For the correct identification of PT, HG and occurring duplications a critical step was the visualization of sulcal boundaries. Three-dimensional surface reconstruction of auditory cortices allowed for reliable allocation of anatomical landmarks (**Fig. 2c**).

Block-designed fMRI was performed during auditory stimulation with different instrumental tones in analogy to MEG stimuli (EPI-sequences, 36 oblique slices parallel to the Sylvian fissure, slice thickness 3 mm, echo time  = 30 ms, repetition time  = 2500 ms). Subsequent to motion correction, alignment and TAL transformation, grouped functional maps were superimposed on a structural T1-weighted data set of one WS individual and the respective 3D-reconstructions of the AC using BrainVoyager software (Brain Innovation QX version 1.8; **Fig. 5**).
